# Lack of Pituitary Adenylate Cyclase–Activating Polypeptide (PACAP) Disturbs Callus Formation

**DOI:** 10.1007/s12031-019-01448-z

**Published:** 2019-12-05

**Authors:** Gergő Józsa, Balázs Dániel Fülöp, László Kovács, Bernadett Czibere, Vince Szegeczki, Tamás Kiss, Tibor Hajdú, Andrea Tamás, Zsuzsanna Helyes, Róza Zákány, Dóra Reglődi, Tamás Juhász

**Affiliations:** 1 Department of Anatomy MTA-PTE PACAP Research Team, Medical School, University of Pécs, Pécs, Hungary; 2 Department of Anatomy, Histology and Embryology, Faculty of Medicine, University of Debrecen, Debrecen, Hungary; 3 Department of Pharmacology and Pharmacotherapy, Medical School, University of Pécs, Pécs, Hungary; 4 Szentágothai Research Centre, Centre for Neuroscience, University of Pécs, Pécs, Hungary

**Keywords:** PACAP, BMP, Collagen type I, ALP, Tibia fracture, Callus

## Abstract

Pituitary adenylate cyclase–activating polypeptide (PACAP) is a naturally secreted signaling peptide and has important regulatory roles in the differentiation of the central nervous system and its absence results in disorders in femur development. PACAP has an important function in prevention of oxidative stress or mechanical stress in chondrogenesis but little is known about its function in bone regeneration. A new callus formation model was set to investigate its role in bone remodeling. Fracturing was 5 mm distal from the proximal articular surface of the tibia and the depth was 0.5 mm. Reproducibility of callus formation was investigated with CT 3, 7, and 21 days after the operation. Absence of PACAP did not alter the alkaline phosphatase (ALP) activation in PACAP KO healing process. In developing callus, the expression of collagen type I increased in wild-type (WT) and PACAP KO mice decreased to the end of healing process. Expression of the elements of BMP signaling was disturbed in the callus formation of PACAP KO mice, as bone morphogenic protein 4 (BMP4) and 6 showed an early reduction in bone regeneration. However, elevated Smad1 expression was demonstrated in PACAP KO mice. Our results indicate that PACAP KO mice show various signs of disturbed bone healing and suggest PACAP compensatory and fine tuning effects in proper bone regeneration.

**Electronic supplementary material:**

The online version of this article (10.1007/s12031-019-01448-z) contains supplementary material, which is available to authorized users.

## Introduction

Bone is one of the hardest tissues of the body and it is responsible for bearing weight and forming a skeleton for movements. Long bones histologically can be divided into compact and spongy bone. Compact bone forms the cortical part of long bones giving a protection for the bone marrow and its thickness plays a role in the weight-bearing stability (Augat et al. 2014). Specific structure and orientation of extracellular matrix molecules have been identified in cortical bone: collagen type I forms concentric lamellas in the osteons, it is spiral in shape and runs perpendicular to the neighboring collagen fibers (Oftadeh et al. 2015). This orientation strongly determines the shape and stability of long bones. In certain gaps of collagen fibers, Ca^2+^ phosphate crystals are embedded forming the inorganic component and giving the mechanical strength of cortical bone (Ascenzi and Roe 2012). Pathological disturbance of appropriate cortical bone formation results in an irregular architecture decreasing the mechanical stability of long bones. Traumatologic long bone fractures and disintegration re-induce proper matrix expression with a well-organized cytokine secretion resulting in a modified but stable matrix architecture and orientation (Hegde et al. 2016). Healing process of cortical bone starts with a local inflammation followed by the formation of callus and remodeling of organic then inorganic matrix (Giganti et al. 2014; Haffner-Luntzer et al. 2017).

Fracture healing is a multi-step process with the activation of intra- and extracellular events from which several molecular mechanisms have been identified as key-regulators of proper regeneration and remodeling (Fazzalari 2011; Giganti et al. 2014). Callus formation of disintegrated bone is initiated by the activation of bone morphogenic proteins (BMPs) which are the most important components of signaling pathways leading to extracellular matrix (ECM) formation (Kloen et al. 2003). BMPs are small cytokines with important functions in the differentiation of different tissues, such as cartilage (Saitta et al. 2018), kidney (Eneman et al. 2016), and bone (Jozsa et al. 2018). They also play an important role in the formation of basal membrane via induction of collagen type IV expression (Reglodi et al. 2018a). BMPs have several subtypes, from which BMP2 is the most important one in bone regeneration (Kloen et al. 2003). BMP4 is also unquestionable in osteogenesis or bone healing (Ou et al. 2015), and it also plays a regulatory role in mesenchymal cell transformation as well as in chondrogenesis (Saitta et al. 2018). BMP5 and BMP6 are believed to be the main BMPs of normal bone formation and maintenance of the cortical bone matrix architecture (Wutzl et al. 2006). Binding of BMPs to their receptors such as BMPR1 can induce the complex formation of Smad transcription factors. Smad1 or 5 has to form a complex with Smad4 which can translocate to the nuclei and triggers ALP expression, proliferation, or collagen expression in osteoblasts (Jozsa et al. 2018). On the other hand, parathyroid hormone (PTH) is a very important substance responsible for activation of intracellular events leading to secrete organic and inorganic components of bone during healing (Milstrey et al. 2017). It has been shown that β-catenin pathway and activation of WNTs are basic signaling connections leading in proper callus formation of the fractured tissues (Yu et al. 2018).

Pituitary adenylate cyclase–activating polypeptide (PACAP) is an evolutionary well-conserved neuropeptide identified in hypothalamo-hypophyseal system (Miyata et al. 1989). Two biological active forms, PACAP 1-38 and PACAP 1-27, exist with a short half-life in vivo (Vaudry et al. 2009). Since its isolation, several peripheral organs have been proven to secrete and release the neuropeptide. PACAP plays a regulatory role in the development of gonads (Reglodi et al. 2018a), cartilage (Juhasz et al. 2014a, 2015b), teeth (Sandor et al. 2014; Fulop et al. 2019), and immune system (Abad and Tan 2018). PACAP has three main G protein coupled receptors, PAC1, VPAC1, and VPAC2, which induce the activation of adenylate cyclase, increase the intracellular cAMP concentration, and trigger PKA phosphorylation activity (Vaudry et al. 2009). Downstream target of this kinase can classically be the CREB transcription factor or Runx2 in osteoblasts (Juhasz et al. 2014b; Jozsa et al. 2018). However, it has been published that PACAP has various signaling crosstalks, like direct connections with the BMP, WNT, hedgehog, or β-catenin signaling pathways (Juhasz et al. 2015a, b). As Runx2, BMP- or WNT-related pathways regulate osteogenesis (Jozsa et al. 2018) and in UMR-106 cell line PACAP has been demonstrated to alter bone differentiation processes (Juhasz et al. 2014b) or its absence alter the micro-architecture of long bones (Jozsa et al. 2018), it comes to the focus of interest whether it has any effects on bone healing processes. It has been demonstrated that the lack of PACAP results in an increased ALP activation and increased organic matrix secretion (Jozsa et al. 2018). On the other hand, PACAP has a protective effect against strong mechanical force in cartilage differentiation (Juhasz et al. 2015b) and it reduces the activity and expression of MMPs during oxidative stress of chondrifying cell cultures (Szentleleky et al. 2019). Based on these facts, PACAP may have a function in regulation of callus formation or bone regeneration processes. Our main goal was to study the effects of PACAP in bone regeneration in a newly developed surgical fracture method.

## Materials and Methods

### Animals

Twenty-four mice were 3-month-old PACAP HZ breeding, and backcrossed with CD1 mice (Hashimoto et al. 2001). Wild-type (WT) (*n* = 12) and PACAP knockout (KO) (*n* = 12) mice were three generation littermates and half of them were females and males. Twelve WT and 12 KO were used for PCR and separately for WB and histology. Mice were fed and watered ad libitum with standard feed pellets and tap water, kept under 12/12 h of light/dark cycles at 20–22 °C and 40–60% humidity. All experiments were approved by the animal ethics committee of the University of Pecs (BA02/2000-24/2011). Genotyping was performed from tail samples with Phire Animal Tissue Direct PCR Kit (Thermo Fischer Scientific, Waltham, MA, USA) (for details, see (Farkas et al. 2017)).

### Induced Fracture and Callus Formation Model

Mice were anesthetized with Euthasol 1% solution 7 μL/10 g by intra-peritoneal injection. Open fracture technique was performed as follows: a 1-cm-incision was made on the anterior aspect of the both proximal parts of the crural region under aseptic surgical technique. Muscles and periosteum were sharply incised. Fracture in the proximal third of tibia was made. The surface of the scalpel was labeled 0.5 mm distance from the cutting edge to ensure the standard depth of the incision. Fracture location was 5 mm distal from the proximal articular surface of the tibia and the depth was 0.5 mm. As the bilateral monocortical anterior osteotomy was stable, the postoperative period did not require any external (plastering) or internal fixatures. Sham-operated animals were used as controls. Wounds were closed using 5/0 nylon suture. Pain management included buprenorphine (0.05–0.1 mg/kg bw) subcutaneously postoperatively for 2 days.

### Light Microscopical Analysis

Both tibias were harvested after sacrificing the mice with an overdose of pentobarbital sodium 3, 7**,** and 21 days after surgery (100 mg/kg bw). Tibias were dissected and additional tissues were removed and washed in PBS three times and fixed in a 4:1 mixture of absolute ethanol and 40% formaldehyde (Sigma-Aldrich MO, USA). Bones were decalcified in 4% EDTA for **4** weeks till bones became soft. Then**,** samples were dehydrated in ascending alcohol row and embedded in paraffin. Five micrometers of serial sections were made. After rehydration**,** hematoxylin**-**eosin (HE, Sigma-Aldrich MO, USA) staining was performed. Staining protocol was carried out according to the manufacturer’s instructions. Photomicrographs were taken using an Olympus DP72 camera on a Nikon Eclipse E800 microscope (Nikon Corporation, Tokyo, Japan).

### Micro-Computed Tomography Measurement

Tibias were scanned by micro-computed tomography (micro-CT) (Skyscan 1176 micro-CT) to confirm fracture and to study the bone healing (callus formation) process. The obtained imaging data were analyzed and reconstructed by two softwares Nrecon and CT Analyzer (Bruker MicroCT, Kontich, Belgium). The scanning parameters used in this study were the following: 55 kV source voltage, 450 μA source current, 350 ms exposure time, 0.5 mm Al filter. Bone structure–related parameters were the bone volume, bone surface, and bone mineral density (BMD).

BMD is defined as the volumetric density of calcium hydroxyapatite (CaHA) in terms of g cm^−3^. It refers to a density measurement restricted to the volume of calcified bone tissue, such as cortical (volumetric) BMD. Hounsfield units (HU) are standard units of X-ray CT density, in which air and water are ascribed values of − 1000 and 0, respectively. It is a useful general CT density calibration system (Lamba et al. 2014). The Skyscan CT analyzer software provides for an integrated calibration of datasets into these two density scales, HU and BMD. Both require the appropriate calibration phantom scans and measurements.

### RT-PCR Reactions

After removing additional tissues from the tibia surface, callus was precisely dissected; approximately 2 mm^3^ sample was removed. Tissues were cryo-ground in liquid nitrogen and dissolved in Trizol (Applied Biosystems, Foster City, CA, USA), and after the addition of 20% RNase-free chloroform, samples were centrifuged at 4 °C at 10,000×*g* for 15 min. Samples were incubated in 500 μL of RNase-free isopropanol at − 20 °C for 1 h; then, total RNA was harvested in RNase-free water and stored at − 20 °C. The assay mixture for reverse transcriptase reaction contained 2 μg RNA, 0.112 μM oligo(dT), 0.5 mM dNTP, and 200 units of High Capacity RT (Applied Biosystems) in 1× RT buffer. For the sequences of primer pairs and further details of polymerase chain reactions, see Table 1. Amplifications were performed in a thermal cycler (Labnet MultiGene™ 96-well Gradient Thermal Cycler; Labnet International, Edison, NJ, USA) in a final volume of 21 μL (containing 1 μL forward and reverse primers [0.4 μM], 0.5 μL dNTP [200 μM], and 5 units of Promega GoTaq® DNA polymerase in 1× reaction buffer) as follows: 95 °C, 2 min, followed by 35 cycles (denaturation, 94 °C, 1 min; annealing at optimized temperatures as given in Table 1 for 1 min; extension, 72 °C, 90 s) and then 72 °C, 10 min. PCR products were analyzed by electrophoresis in 1.2% agarose gel containing ethidium bromide. The figures show one representative lane-row from the same experiment for the better comparison. Actin was used as internal control. Optical density of signals was measured by using ImageJ 1.40 g freeware and results were normalized to the optical density of control tissue. Diagrams show the statistical analysis of the experiments where every separated experiment (at least 3) was normalized for its own actin signal and then statistical differences were calculated. Detailed comparison has been done to normalized and investigate significant differences between separate groups. Results are seen in Supplementary Figure 1.

**Table 1:** Nucleotide sequences, amplification sites, GenBank accession numbers, amplimer sizes, and PCR reaction conditions for each primer pair are shown

Gene	Primer	Nucleotide sequence (5′ → 3′)	GenBank ID	Annealing temperature	Amplimer size (bp)
Alkaline phosphatase (Alpl)	Sense	GAA GTC CGT GGG CAT CGT (474–491)	NM_013059	59 °C	347
	Antisense	CAG TGC GGT TCC AGA CAT AG (801–820)			
BMP2 (Bmp2)	Sense	AAG CCA GGT GTC TCC AAG (697–714)	NM_017178.1	53 °C	209
	Antisense	AAG TCC ACA TAC AAA GGG TG (886–905)			
BMP4 (Bmp4)	Sense	TAG TCC CAA GCA TCA CCC (876–893)	NM_012827.2	53 °C	294
	Antisense	TCG TAC TCG TCC AGA TAC AAC (1149–1169)			
BMP6 (Bmp6)	Sense	CCC AGA TTC CTG AGG GTG A (936–954)	NM_013107.1	56 °C	248
	Antisense	CAT GTT GTG CTG CGG TGT (1166–1183)			
Collagen type I (Col1a1)	Sense	GGG CGA GTG CTG TGC TTT (348–365)	NM_007742.3	60 °C	388
	Antisense	GGG ACC CAT TGG ACC TGA A (717–735)			
Actin (Actb)	Sense	GCC AAC CGT GAA AAG ATG A (419–437)	NM_007393.5	54 °C	462
	Antisense	CAA GAA GGA AGG CTG GAA AA (861–880)			
Smad1 (Smad1)	Sense	AGC ACC TAC CCT CAC TCC C (935–953)	NM_013130.2	56 °C	306
	Antisense	GAA ACC ATC CAC CAA CAC G (1222–1240)			

### Western Blot

After removing additional tissues from the tibia surface, callus was precisely dissected and a sample of approximately 2 mm^3^ in size was removed. Tissues were cryo-ground in liquid nitrogen. After centrifugation, tissue pellets were suspended in 100 μL of homogenization RIPA. Samples were stored at − 70 °C. Suspensions were sonicated by pulsing burst for 30 s at 40 A (Cole-Parmer, IL, USA). For Western blotting, total cell lysates were used. Samples for SDS-PAGE were prepared by the addition of Laemmli electrophoresis sample buffer (4% SDS, 10% 2-mercaptoethanol, 20% glycerol, 0.004% bromophenol blue, 0.125 M TrisHCl pH 6.8) to cell lysates to set equal protein concentration of samples, and boiled for 10 min. About 20 μg of protein was separated by 7.5% SDS-PAGE gel for detection of ALP, collagen type I, BMP2, BMP4, BMP6, and Smad1. Proteins were transferred electrophoretically to nitrocellulose membranes. After blocking with 5% non-fat dry milk in phosphate-buffered saline (PBST) with 0.1% Tween 20, membranes were washed and exposed to the primary antibodies overnight at 4 °C in the dilution as given in Table 2. After washing for 30 min in PBST, membranes were incubated with anti-rabbit IgG (Bio-Rad Laboratories, CA, USA) in 1:1500, anti-goat IgG (Sigma-Aldrich MO, USA) in 1:2000, and anti-mouse IgG (Bio-Rad Laboratories, CA, USA) in 1:1500 dilution. Signals were detected by enhanced chemiluminescence (Pierce™, MA, USA) according to the instructions of the manufacturer. Signals were developed with on X-ray films and documented by gel documentary system (Fluorchem E, ProteinSimple, CA, USA). The figures show one representative lane-row from the same experiment. Optical density of Western blot signals was measured by using ImageJ 1.40 g freeware and results were normalized to that of control samples. Diagrams show the statistical analysis of the experiments where every separated experiment (at least 3) was normalized for its own actin signal and then statistical differences were calculated. Detailed comparison has been done to investigate significant differences between separate subgroups. Results are seen in Supplementary Figure 1.

**Table 2:** Tables of antibodies used in the experiments

Antibody	Host animal	Dilution	Distributor
Anti-Coll. I.	Mouse, monoclonal	1:1000	Sigma-Aldrich, St. Louis, MO, USA
Anti-ALP	Rabbit, polyclonal	1:500	Abcam, Camridge, UK
Anti-BMP2	Mouse, monoclonal	1:500	Abcam, Camridge, UK
Anti-BMP4	Rabbit, polyclonal	1:600	Cell Signaling, Danvers, MA, USA
Anti-BMP6	Rabbit, polyclonal	1:200	Santa Cruz Biotechnology Inc., Santa Cruz, CA, USA
Anti-Smad1	Rabbit, polyclonal	1:600	Cell Signaling, Danvers, MA, USA
Anti-Actin	Mouse, monoclonal	1:10000	Sigma-Aldrich, St. Louis, MO, USA

### Statistical Analysis

All data are representative of at least four independent experiments; data are mean values. Statistical significances between controls (sham-operated sides) and operated sides were determined by one-way analysis of variance (ANOVA), followed by Tukey’s HSD post hoc test.

## Results

### Fracture Technique

**Fig. 1:**
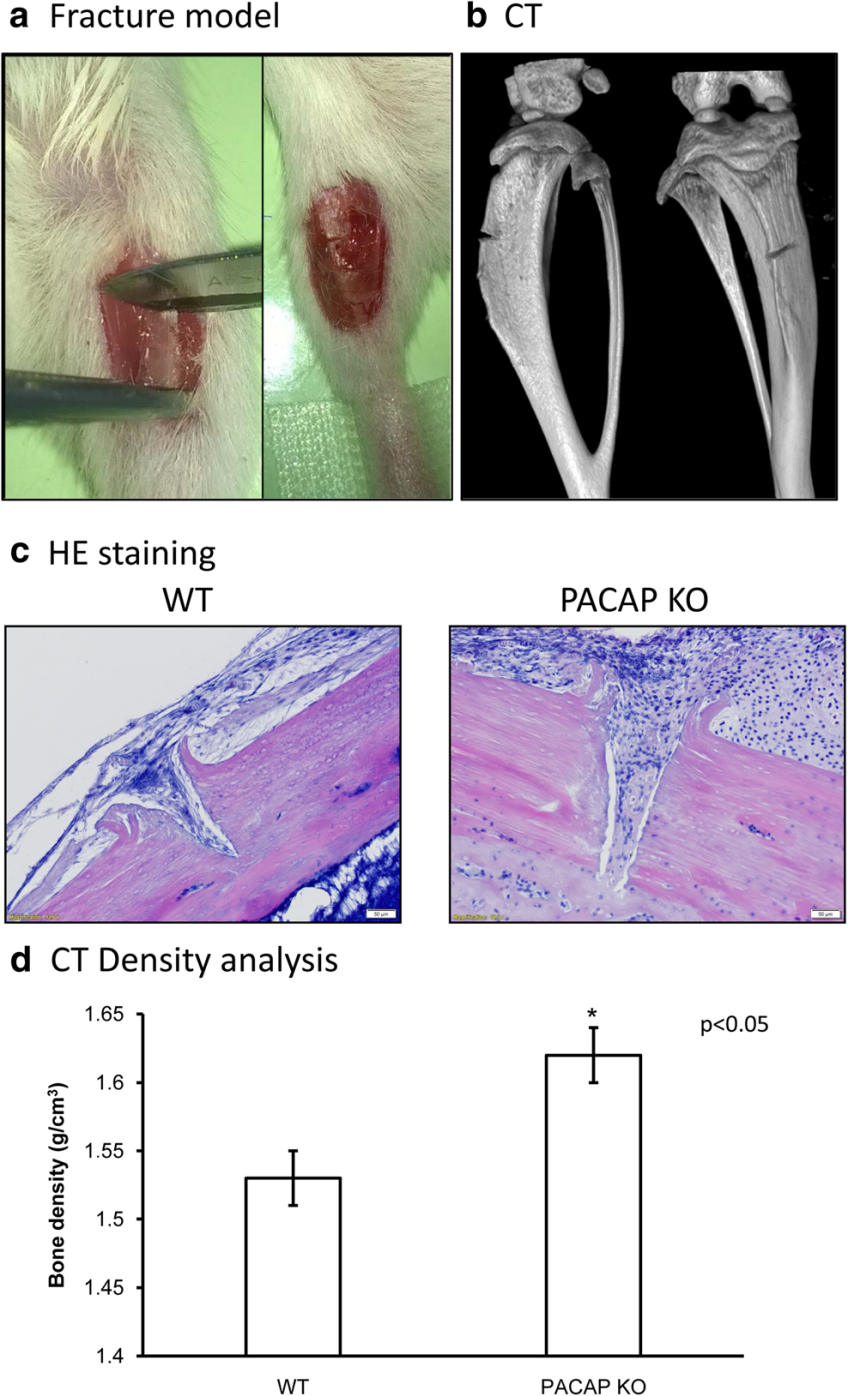
Morphological analysis of tibia callus formation of wild-type (WT) and pituitary adenylate cyclase–activating polypeptide (PACAP) knockout (KO) mice. Surgical operation of tibia (**a**) for demonstration of fracture and the precise position and depth of incision. Scanning of fractured tibia with micro-CT (**b**) to demonstrate the location and depth of the fracture. Hematoxylin-eosin (HE) staining (**c**) to visualize the histological morphology of fractures. Original magnification was × 20. Scale bar, 50 μm. CT analysis (**d**) of mouse tibia. Representative data of 12 independent experiments. Asterisks indicate significant (*p < 0.05) difference of callus densities in PACAP KO animals compared to the WTs

For the investigation of callus formation, we developed a new surgical method which was well reproducible and showed callus formation in a standard way. With surgical scalpel, we made an incision 5 mm from the condyle of the tibia on the anterior margin of the limb. With a modified scalpel, we made a 0.5 mm deep fracture (Fig. 1a). The position of the incision was measurable and precisely reproducible. First, we analyzed the fracture position and depth with micro-CT, which supported the reproducibility of the method and also showed a standard bone fracture on the anterior margin of the tibia in all cases (Fig. 1b). Both in PACAP KO and WT mice, the cortical part of the tibial anterior margin showed discontinuity and on the third day of the healing process callus formation was shown (Fig. 1c). CT analysis, performed on day 3 of healing, detected significant differences between WT and PACAP animals. The density of PACAP KO callus was higher than in WT mice (Fig. 1d).

### ALP Expression in Callus Formation

**Fig. 2:**
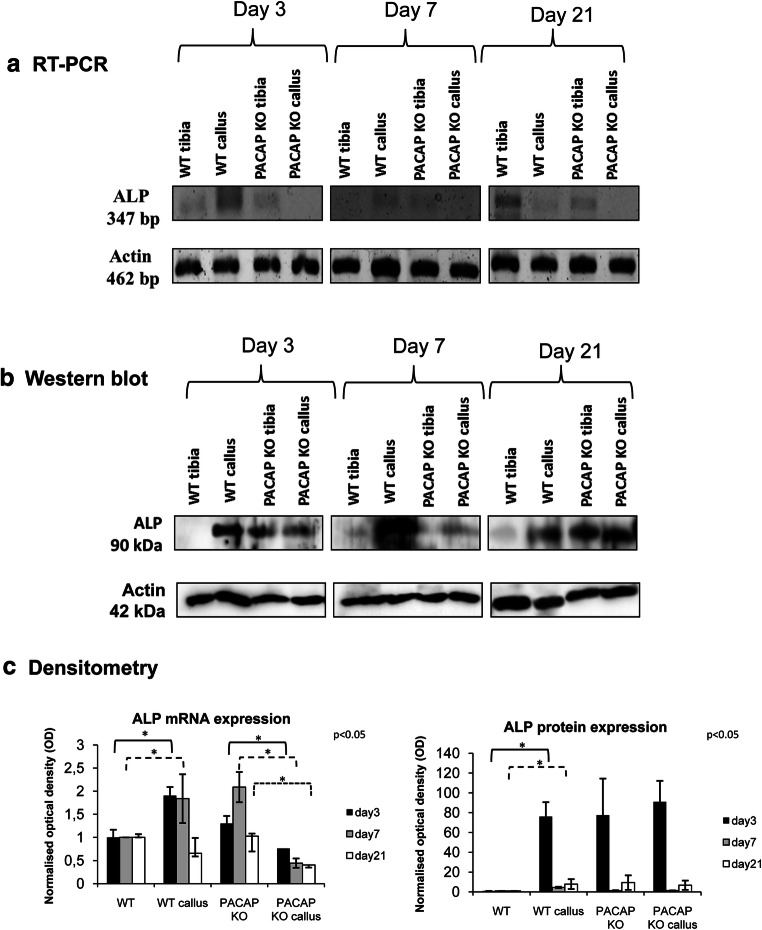
mRNA (**a**) and protein (**b**) expression of alkaline phosphatase (ALP). For RT-PCR and Western blot reactions, Actin was used as inner control. Representative data of 4 independent experiments on days 3, 7, and 21 of callus formation. Optical density (**c**) of signals was measured and results were normalized to the optical density of WT and PACAP sham-operated tibia. For diagrams **a** and **b**, densities and means of four independent experiments (±standard error of the mean) are shown in the figures. Asterisks indicate significant differences compared to sham-operated sides (*p* < 0.05; one-way ANOVA followed by Tukey’s HSD test)

First, we analyzed the inorganic matrix production during callus formation. As ALP is one of the most important factors inducing matrix component production, we followed its expression on 3 different days of bone regeneration. As the size of the callus was relatively small, only weak signals appeared in the experiments. On day 3 of callus formation, mRNA expression of ALP was barely detected in control WT samples but showed an elevated ALP mRNA expression in WT callus (Fig. 2a, c, Supp. Fig. 1A). On the other hand, we demonstrated a higher ALP mRNA in PACAP KO tibia compared with PACAP KO callus samples (Fig. 2a, c, Supp. Fig. 1A). Similar expression pattern was detected on day 7 of callus formation (Fig. 2a, c, Supp. Fig. 1A). Reduced ALP expression was detected in callus of WT animals at the end of the healing process (Fig. 2a, c, Supp. Fig. 1A). In PACAP KO samples, decreased mRNA expressions were shown on different days of callus formation (Fig. 2a, c, Supp. Fig. 1A).

Interestingly, protein expression of ALP was not in correlation with its mRNA expression. In WT tibias, almost undetectable ALP protein level could be seen (Fig. 2b, c, Supp. Fig. 1B), while in PACAP KO tibia, higher expression of ALP was detected (Fig. 2b, c, Supp. Fig. 1B). Callus formation increased the ALP protein expression on days 3 and 7 (Fig. 2b, c) and started to reduce at 21st day of healing in WT mice (Fig. 2b, c, Supp. Fig. 1B). On the contrary, no elevation in protein expression was detected in PACAP KO callus on days 3, 7, and 21 of healing process (Fig. 2b, c, Supp. Fig. 1B).

### Organic Matrix Production in Bone Fracture Healing Processes

**Fig. 3:**
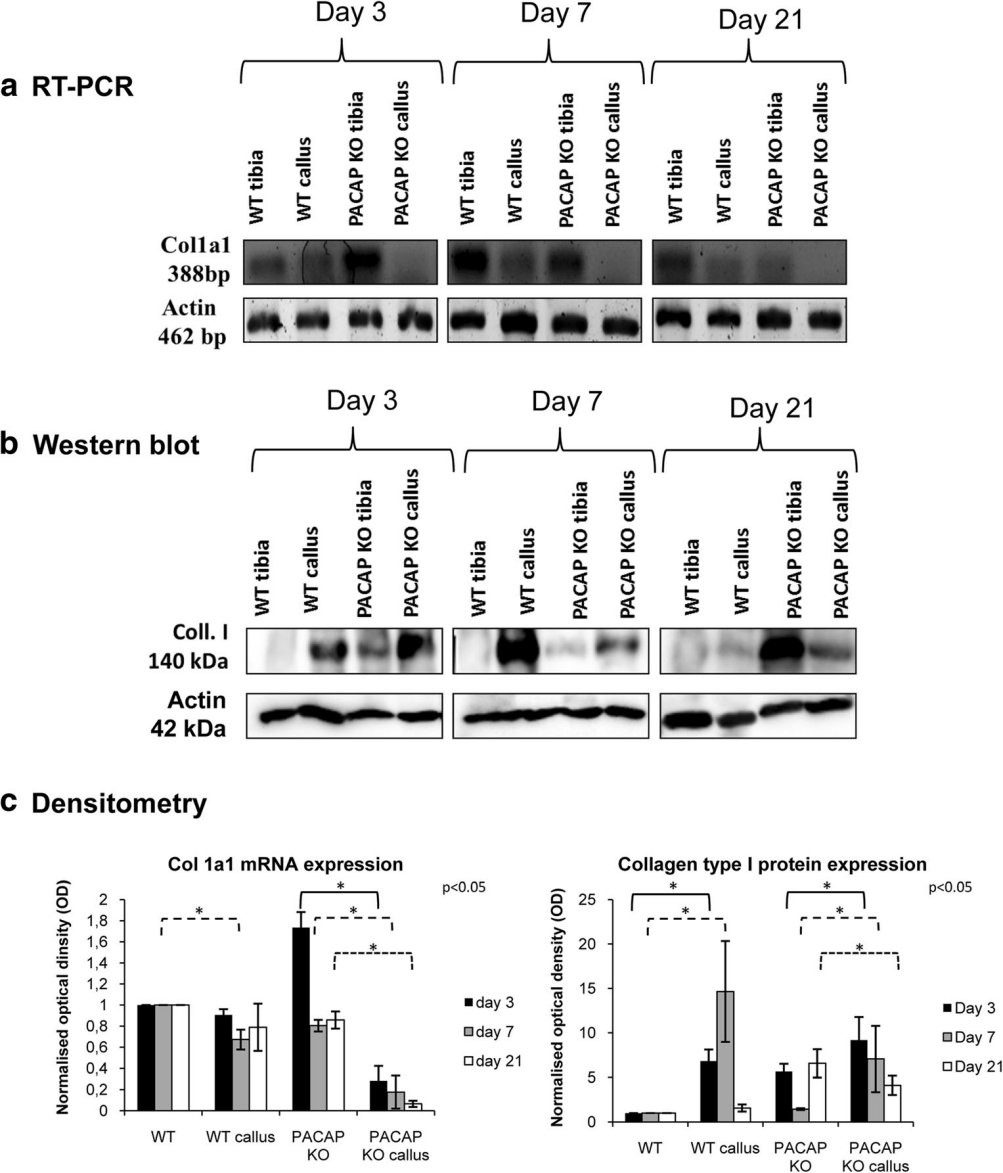
mRNA (**a**) and protein (**b**) expression of collagen type I. For RT-PCR and Western blot reactions, Actin was used as inner controls. Representative data of 4 independent experiments on days 3, 7, and 21 of callus formation. Optical density (**c**) of signals was measured and results were normalized to the optical density of WT tibia. For diagrams **a** and **b**, densities and means (±standard error of the mean) are shown in the figures. Asterisks indicate significant differences compared to sham-operated sides (*p* < 0.05; one-way ANOVA followed by Tukey’s HSD test)

Proper collagen type I production is also necessary for callus formation; therefore, we analyzed its expression. mRNA expression of collagen type I was low in non-fractured tibia and in callus of WT mice, with decreased expression on days 7 and 21 (Fig. 3a, c, Supp. Fig. 1C). As we have previously described (Jozsa et al. 2018), PACAP KO mice have higher collagen type I mRNA expression compared to WT (Fig. 3a, c, Supp. Fig. 1C). Similarly to WT mice, lower, occasionally undetectable, collagen expression was shown at all investigated days of callus formation in KO mice (Fig. 3a, c, Supp. Fig. 1C).

Protein expression of collagen type I was not in correlation with its mRNA expression. Low expression was detected in WT animals (Fig. 3b, c, Supp. Fig. 1D). On day 3, elevated expression was detected, peaking on day 7 and decreased to day 21 of callus formation (Fig. 3b, c, Supp. Fig. 1D). Expression of collagen type I was elevated in PACAP KO mice compared with WT controls, showing a further elevation on day 3 (Fig. 3b, c, Supp. Fig. 1D). A lower increase was shown in PACAP KO mice on day 7, compared with the callus of WT animals (Fig. 3b, c, Supp. Fig. 1D). To day 21 of callus formation, it was significantly reduced but remained higher than in callus samples of WT rodents (Fig. 3b, c, Supp. Fig. 1D).

### BMP Signaling Alteration in PACAP KO Callus Formation

**Fig. 4:**
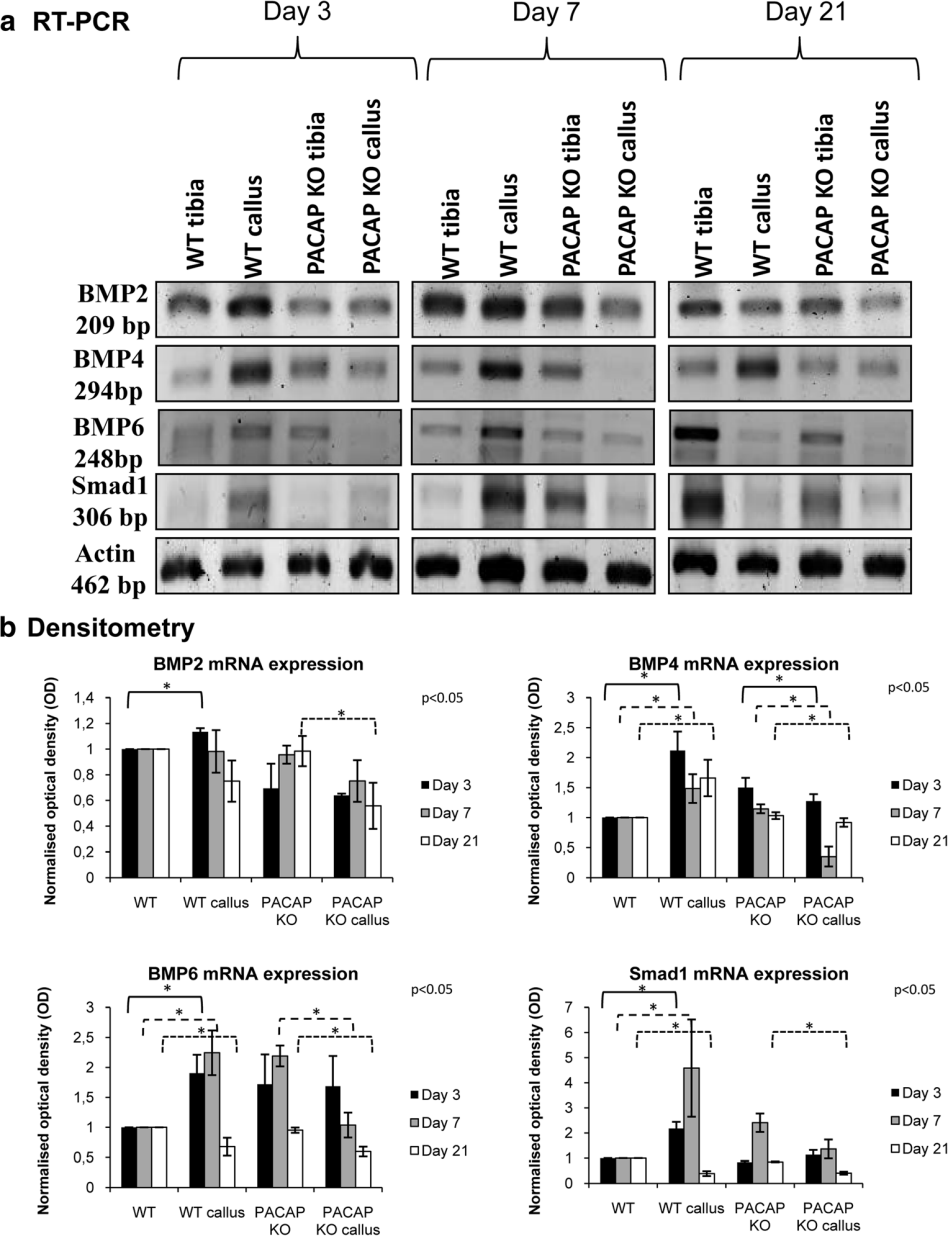
mRNA (**a**) expressions of BMP2, 4, and 6 and Smad1. For RT-PCR reactions, Actin was used as inner control. Representative data of 4 independent experiments on days 3, 7, and 21 of callus formation. Optical density (**b**) of signals was measured and results were normalized to the optical density of WT tibia. For diagram **a**, densities and means of four independent experiments (±standard error of the mean) are shown in the figures. Asterisks indicate significant differences compared to sham-operated sides (*p* < 0.05; one-way ANOVA followed by Tukey’s HSD test)

As BMPs are among the most important factors in callus formation and BMP signaling has direct connection with PACAP signalization, we investigated different elements of BMP signaling. mRNA expression of BMP2 was elevated on day 3 of callus formation in WT animals (Fig. 4a, b, Supp. Fig. 1E) but started to decrease from day 7 and reduced to day 21 of healing (Fig. 4a, b, Supp. Fig. 1E). In PACAP KO mice, no alteration was detected in samples on days 3 and 7 but significant decrease was shown on day 21 (Fig. 4a, b, Supp. Fig. 1E). BMP4 mRNA expression increased on all investigated days in WT animals (Fig. 4a, b, Supp. Fig. 1G). On the contrary, slight reduction was visible in PACAP KO callus compared with the PACAP KO control tibia and WT callus formation (Fig. 4a, b, Supp. Fig. 1G). Weak BMP6 signals appeared in WT samples with and increased intensity in callus samples on days 3 and 7 (Fig. 4a, b, Supp. Fig. 1I). On day 21, BMP6 expression decreased to the level of WT bone (Fig. 4a, b, Supp. Fig. 1I). On the other hand, significant decrease was detected on days 7 and 21 in PACAP KO mice compared with the sham-operated side (Fig. 4a, b, Supp. Fig. 1I). Smad1 mRNA expression increased in WT callus for days 3 and 7 and then decreased for the end of callus formation (Fig. 4a, b, Supp. Fig. 1K). In PACAP KO callus, some elevations were detected only on day 3 but reduced signal was detected on days 7 and 21 (Fig. 4a, b, Supp. Fig. 1K).

**Fig. 5:**
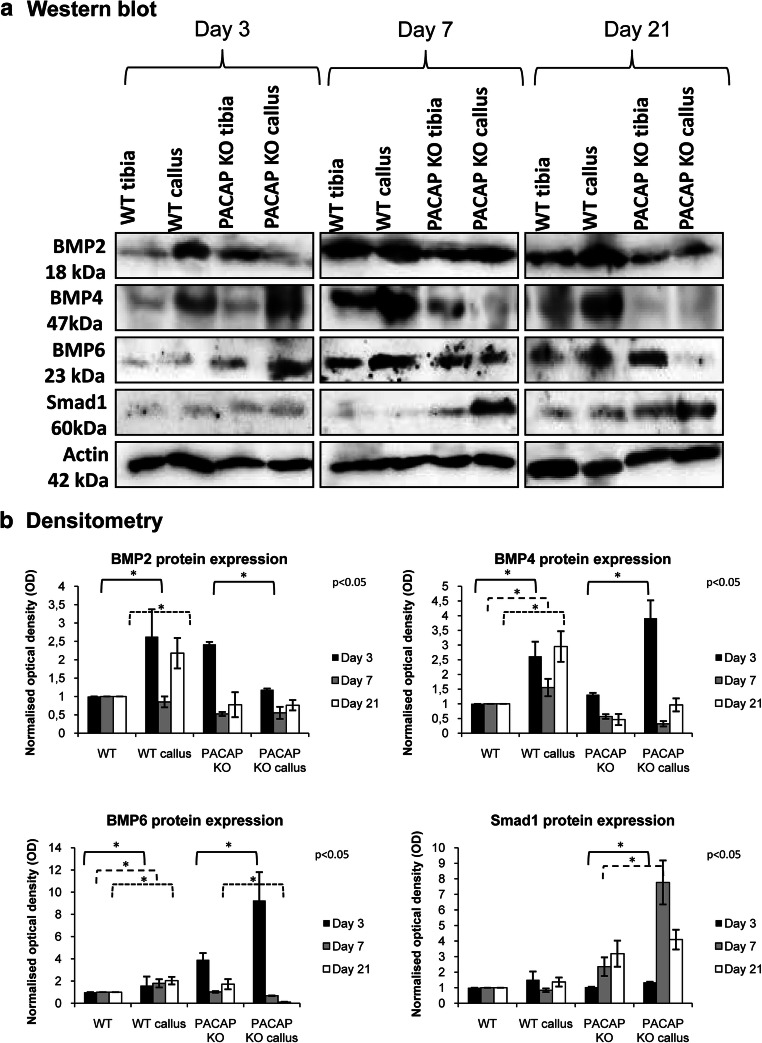
Protein (**a**) expression of expressions of BMP2, 4, and 6 and Smad1. For Western blot reactions, Actin was used as inner control. Representative data of 4 independent experiments on days 3, 7, and 21 of callus formation. Optical density (**b**) of signals was measured and results were normalized to the optical density of WT tibia. For diagram **a**, densities and means of four independent experiments (± standard error of the mean) are shown in the figures. Asterisks indicate significant differences compared to sham-operated sides (*p* < 0.05; one-way ANOVA followed by Tukey’s HSD test)

BMP2 protein expression was increased on days 3 and 21 in WT callus samples with no changes on day 7 of healing. On the contrary, a significant decrease was detected on day 3 in PACAP KO callus compared with the untreated bone, while no alterations were detected on days 7 and 21 (Fig. 5a, b, Supp. Fig. 1F). BMP4 protein expression was elevated in WT and PACAP KO callus on day 3 (Fig. 5a, b, Supp. Fig. 1H). On days 7 and 21, increased BMP4 protein expression was detected in callus samples of WT animals, but a significant elevation was detected in PACAP KO mice only on day 3, with no alterations on days 7 and 21 (Fig. 5a, b, Supp. Fig. 1H). BMP6 protein expression showed a significant elevation on the fractured side on the 3 investigated days in WT mice (Fig. 5a, b, Supp. Fig. 1J). On the contrary, an elevation was detected on day 3 followed by a significant reduction to end of regeneration in PACAP KO mice (Fig. 5a, b, Supp. Fig. 1J). Downstream target of BMP signaling is Smad1, which did not alter in WT callus samples, but showed a strong increase in PACAP KO rodents at the end of bone regeneration (Fig. 5a, b, Supp. Fig. 1L).

## Discussion

Although PACAP KO mice do not show marked macroscopical alterations, several changes have been detected in the absence of PACAP in different tissues during development, such as in teeth (Sandor et al. 2014, 2016; Fulop et al. 2019), testes (Reglodi et al. 2018a), and ovaries (Reglodi et al. 2012). Lack of PACAP also influences behavior of these animals (Farkas et al. 2017), and results in alterations of neuronal development (Rivnyak et al. 2018). Not only the absence of the neuropeptide but its addition also affects the differentiation of chondrocytes (Juhasz et al. 2014a), neurons (Guo et al. 2016), and mammary epithelial cells (Csanaky et al. 2014). We have demonstrated earlier that PACAP increases Ca^2+^ release in osteoblasts and induces their differentiation (Juhasz et al. 2014b). Further investigation of PACAP KO mice femur showed several signaling alterations and morphological changes (Jozsa et al. 2018). Femur cortical thickness and diameter of its bone marrow cavity were altered in these animals. Moreover, collagen type I secretion and ALP expression and activation elevated in PACAP KO femurs (Jozsa et al. 2018). The importance of PACAP in various kinds of stress has been detected recently including compensation of the harmful effects of oxidative stress and mechanical load in chondrogenesis (Juhasz et al. 2014a, 2015b). It decreases the activity of matrix degrading enzymes in chondrogenic cell cultures during mechanical overload (Szentleleky et al. 2019). It also has a protective function in retinopathy of prematurity (Kvarik et al. 2016) and ischemic conditions (Reglodi et al. 2018b). All of these facts indicate that PACAP can have important functions in fractured bone remodeling and healing.

Investigation of callus formation in mouse models is widely used to follow bone healing with different modes of fracture induction (Haffner-Luntzer et al. 2017; Fujisawa et al. 2018; Ota et al. 2018). The closed tibia fracture model is well described and involves the use of stainless steel fixation pins. Disadvantages of this model are the lack of both axial and rotational stability with using a pin, the high risk of knee dislocation, and the potential for intramedullary cavity damage (Schindeler et al. 2008). For standardization of callus position, we developed a well-reproducible surgical fracture method. We used 24 animals from WT and PACAP KO groups and analyzed callus formation with CT and histological staining, which showed a precisely positioned callus formation. This way, callus samples can be removed from the bone and standardized comparability with molecular biological methods is guaranteed.

Activation of different factors is important during bone regeneration to induce proper organic and inorganic matrix production. One of the best indicators of callus formation is the increased ALP expression and activation (Yu et al. 2018). PACAP affects ALP function (Juhasz et al. 2014b) and its lack results in an increased ALP expression and activation in femurs of PACAP KO mice (Jozsa et al. 2018). Interestingly, significant mRNA reduction was detected in PACAP KO callus samples. Similarly to our previous results, we hypothesized that PACAP influenced post-transcriptional processes (Juhasz et al. 2014a, 2015b). In normal callus formation of WT animals, ALP protein level continuously decreased to the end of bone regeneration, similarly to normal callus formation of dogs (Komnenou et al. 2005). On the contrary, the higher ALP level in PACAP KO animals did not reduce to the end of bone healing, indicating a disturbed callus formation in PACAP KO mice. Togari et al. have proven that different neuropeptides and their receptors have an effect on ALP function and bone metabolism (Togari et al. 1997). In our previous study, we have shown that the lack of PACAP increases the expression and activity of ALP and results in focal Ca^2+^ salt accumulation (Jozsa et al. 2018). This ALP overexpression can be detected in PACAP KO tibia callus without showing an alteration in expression. The possible reason is that ALP can be regulated PACAP dependently and independently as it was discussed by (Jozsa et al. 2018). In this study, we provided additional data that PACAP has a fine tuning function in proper bone regeneration, which can be a direct effect through PKA-Runx2-ALP axis (Jozsa et al. 2018) or indirect via ß-catenin-WNT pathways (Yu et al. 2014).

Formation of collagen type I, part of the organic matrix, was disturbed in PACAP KO bone (Jozsa et al. 2018), with a significant elevation resulting in thickened collagen lamellas in the osteons (Jozsa et al. 2018). Expression of collagen type I showed a peak and its expression normalized to the end of bone formation in normal callus formation (Joerring et al. 1994). Similar results were detected in our model in WT mice, but collagen expression in PACAP KO mice was significantly higher during callus formation. This result also indicates an abnormal callus formation with a softened and organic matrix rich callus composition. Alteration of WNT pathways is proven to change proper collagen secretion and results in osteoporosis (Schulze et al. 2010). As mentioned above, WNT-ß-catenin pathway has a crosstalk with PACAP signaling (Yu et al. 2014), which further strengthens the hypothesis that PACAP regulation has a balancing function in proper collagen secretion. Furthermore, lack of PACAP shifts the ECM production towards the organic matrix expression, altering proper bone healing process.

As BMPs are important indicators of callus formation, we focused on the expression of BMP2, 4, and 6, which have important functions in bone remodeling processes (Li et al. 2015; Das et al. 2016). BMPs have been shown to have direct connections with PACAP signaling (Laszlo et al. 2019). Furthermore, we also detected BMP alterations in PACAP KO femurs (Jozsa et al. 2018). Interestingly, a significant reduction was detected in their mRNA expression suggesting PACAP’s post-transcriptional effects (Juhasz et al. 2014a, 2015b). BMP2 protein expression was reduced in PACAP KO callus (Jozsa et al. 2018), but significant elevations were detected in its expression in WT callus. BMP4 protein expression was higher in WT groups in bone healing but an early decrease was detected in PACAP KO mice callus. BMP6 is one of the BMPs which has an important function in mature bone homeostasis and in healing (Beederman et al. 2013). In WT callus, expression of BMP6 continuously increased but an early reduction was detected in PACAP KO callus, followed by a strong decrease at the end of bone regeneration. On the contrary, Smad1 expression was elevated to the termination of bone healing. These results also suggest an active but PACAP-BMP independent Smad1 activation during bone regeneration (Juhasz et al. 2014b; Jozsa et al. 2018). Lack of PACAP disturbs the normal BMP release and enhances the early regeneration via BMP4 and 6 expression. Accumulation of these cytokines is not stimulated in the lack of PACAP and results in a delayed and PACAP independent triggering of Smad activation. These data support the indirect fine tuning function of PACAP on BMP signaling, controlling proper callus formation and regeneration.

In summary, in our study, we developed a precisely reproducible fracture method that enables standardized molecular analysis. We provided evidence that PACAP has an important function in proper bone regeneration. The lack of the neuropeptide disturbs normal organic and inorganic matrix production and alters normal BMP activation in callus formation.

## Electronic supplementary material

ESM 1 (PDF 278 kb)

## Abbreviations

BMP: bone morphogenetic protein
cAMP: cyclic adenosine monophosphate
CREB: cAMP response element-binding protein
dNTP: deoxynucleotide triphosphate
ECM: extracellular matrix
EDTA: ethylene diamine tetra-acetic acid
EGTA: ethylene glycol-bis(β-aminoethyl ether)-N,N,N′,N′-tetra-acetic acid
FGF: fibroblast growth factor
HEPES: 4-(2-hydroxyethyl)-1-piperazineethanesulfonic acid
MAPK: mitogen-activated protein kinase
PAC1: pituitary adenylate cyclase–activating polypeptide type I receptor
PACAP: pituitary adenylate cyclase–activating polypeptide
PBS: phosphate-buffered saline
PBST: phosphate-buffered saline supplemented with 1% Tween-20
PKA: protein kinase A
PKC: protein kinase C
RT-PCR: reverse transcription followed by polymerase chain reaction
Runx2: runt-related transcription factor 2
Smad1: SMA (“small” worm phenotype) and drosophila MAD (“mothers against decapentaplegic”)
TGFβ: transforming growth factor-β
VIP: vasoactive intestinal polypeptide
VPAC: vasoactive intestinal polypeptide receptor
WNT: wingless int1
