# Supplementary material for: Lack of Pituitary Adenylate Cyclase–Activating Polypeptide (PACAP) Disturbs Callus Formation
Source: J Mol Neurosci. 2019 Dec 5;71(8):1543–55. doi: 10.1007/s12031-019-01448-z (PMC8349325; doi:10.1007/s12031-019-01448-z)
Supplement: Supplementary file 1 — (PDF 278 kb) [file 12031_2019_1448_MOESM1_ESM.pdf]

Lack of pituitary adenylate cyclase activating polypeptide (PACAP) disturbs callus formation

Gergő Józsa[1], Balázs Fülöp[1], László Kovács[1], Bernadett Czibere[2], Vince Szegeczki[2], Tamás Kiss[3], Tibor Hajdú[2], Andrea Tamás[1], Zsuzsanna Helyes[3], Róza Zákány[2], Dóra Reglődi[1#], Tamás Juhász[2*#]

Supplementary Figures

Statistical Analysis

All data are representative of at least three independent experiments. Where applicable, data are expressed as mean ± SEM. Statistical analysis was performed by One Way ANOVA test combined with post hoc tests. Where ANOVA reported significant differences between the groups ($p<0.05$) a post hoc test (multiple comparison versus control group, Dunnett's method) was used to isolate the groups that differed from the control group at $p<0.05$. The respective control group was the sham operated WT control when comparison was made between WT control, WT callus, PACAP KO, and PACAP KO callus groups, whereas the WT callus was used as control in the post hoc test when comparison was made between WT callus, PACAP KO and PACAP KO callus samples. Statistical analysis and comparison of Western blot and PCR results can be seen in Supplementary Figs 1A, 1B, 1C, 1D, 1E, 1F, 1G, 1H, 1I, 1J, 1K and 1L.

Supplementary Figure 1. For RT-PCR and Western blot reactions, Actin was used as control. Optical density of signals was measured and results were normalised to the optical density of controls. In tables "+" represent significant differences between integrated densities of signals determined by ImageJ software.

ALP PCR Day 3	WT	WT callus	PACAP KO	PACAP KO callus
WT		+		
WT callus			+	+
PACAP KO				+
PACAP KO callus				

Supp Fig 1A

ALP PCR Day 7	WT	WT callus	PACAP KO	PACAP KO callus
WT		+	+	
WT callus				+
PACAP KO				+
PACAP KO callus				

ALP PCR Day 21	WT	WT callus	PACAP KO	PACAP KO callus
WT				+
WT callus				
PACAP KO				+
PACAP KO callus				

Supp Fig 1B

ALP WB Day 3	WT	WT callus	PACAP KO	PACAP KO callus
WT		+	+	+
WT callus				
PACAP KO				
PACAP KO callus				

ALP WB Day 7	WT	WT callus	PACAP KO	PACAP KO callus
WT		+		
WT callus			+	+
PACAP KO				
PACAP KO callus				

ALP WB Day 21	WT	WT callus	PACAP KO	PACAP KO callus
WT				
WT callus				
PACAP KO				
PACAP KO callus				

Supp Fig 1C

Col I PCR Day 3	WT	WT callus	PACAP KO	PACAP KO callus
WT			+	+
WT callus			+	+
PACAP KO				+
PACAP KO callus				

Col I PCR Day 7	WT	WT callus	PACAP KO	PACAP KO callus
WT		+		+
WT callus				+
PACAP KO				+
PACAP KO callus				

Col I PCR Day 21	WT	WT callus	PACAP KO	PACAP KO callus
WT				+
WT callus				+
PACAP KO				+
PACAP KO callus				

Supp Fig 1D

Col I WB Day 3	WT	WT callus	PACAP KO	PACAP KO callus
WT		+	+	+
WT callus				
PACAP KO				
PACAP KO callus				

Col I WB Day 7	WT	WT callus	PACAP KO	PACAP KO callus
WT		+		
WT callus			+	
PACAP KO				
PACAP KO callus				

Col I WB Day 21	WT	WT callus	PACAP KO	PACAP KO callus
WT			+	+
WT callus			+	
PACAP KO				
PACAP KO callus				

Supp Fig 1E

BMP2 PCR Day 3	WT	WT callus	PACAP KO	PACAP KO callus
WT			+	+
WT callus			+	+
PACAP KO				
PACAP KO callus				

BMP2 PCR Day 7	WT	WT callus	PACAP KO	PACAP KO callus
WT				
WT callus				
PACAP KO				
PACAP KO callus				

BMP2 PCR Day 21	WT	WT callus	PACAP KO	PACAP KO callus
WT				+
WT callus				
PACAP KO				+
PACAP KO callus				

Supp Fig 1F

BMP2 WB Day 3	WT	WT callus	PACAP KO	PACAP KO callus
WT		+	+	
WT callus				+
PACAP KO				+
PACAP KO callus				

BMP2 WB Day 7	WT	WT callus	PACAP KO	PACAP KO callus
WT			+	+
WT callus			+	
PACAP KO				
PACAP KO callus				

BMP2 WB Day 21	WT	WT callus	PACAP KO	PACAP KO callus
WT		+		
WT callus			+	+
PACAP KO				
PACAP KO callus				

Supp Fig 1G

BMP4 PCR Day 3	WT	WT callus	PACAP KO	PACAP KO callus
WT		+	+	
WT callus			+	+
PACAP KO				
PACAP KO callus				

BMP4 PCR Day 7	WT	WT callus	PACAP KO	PACAP KO callus
WT		+		+
WT callus				+
PACAP KO				+
PACAP KO callus				

BMP4 PCR Day 21	WT	WT callus	PACAP KO	PACAP KO callus
WT		+		
WT callus			+	+
PACAP KO				
PACAP KO callus				

Supp Fig 1H

BMP4 WB Day 3	WT	WT callus	PACAP KO	PACAP KO callus
WT		+		+
WT callus			+	+
PACAP KO				+
PACAP KO callus				

BMP4 WB Day 7	WT	WT callus	PACAP KO	PACAP KO callus
WT		+	+	+
WT callus			+	+
PACAP KO				
PACAP KO callus				

BMP4 WB Day 21	WT	WT callus	PACAP KO	PACAP KO callus
WT		+		
WT callus			+	+
PACAP KO				
PACAP KO callus				

Supp Fig 1I

BMP6 PCR Day 3	WT	WT callus	PACAP KO	PACAP KO callus
WT				
WT callus				
PACAP KO				
PACAP KO callus				

BMP6 PCR Day 7	WT	WT callus	PACAP KO	PACAP KO callus
WT		+	+	
WT callus				+
PACAP KO				+
PACAP KO callus				

BMP6 PCR Day 21	WT	WT callus	PACAP KO	PACAP KO callus
WT		+		+
WT callus			+	
PACAP KO				+
PACAP KO callus				

Supp Fig 1J

BMP6 WB Day 3	WT	WT callus	PACAP KO	PACAP KO callus
WT				+
WT callus				+
PACAP KO				+
PACAP KO callus				

BMP6 WB Day 7	WT	WT callus	PACAP KO	PACAP KO callus
WT		+		
WT callus			+	+
PACAP KO				
PACAP KO callus				

BMP6 WB Day 21	WT	WT callus	PACAP KO	PACAP KO callus
WT		+		+
WT callus				+
PACAP KO				+
PACAP KO callus				

Supp Fig 1K

Smad1 PCR Day 3	WT	WT callus	PACAP KO	PACAP KO callus
WT		+		
WT callus			+	+
PACAP KO				
PACAP KO callus				

Smad1 PCR Day 7	WT	WT callus	PACAP KO	PACAP KO callus
WT		+		
WT callus				+
PACAP KO				
PACAP KO callus				

Smad1 PCR Day 21	WT	WT callus	PACAP KO	PACAP KO callus
WT		+		+
WT callus			+	
PACAP KO				+
PACAP KO callus				

Supp Fig 1L

Smad1 WB Day 3	WT	WT callus	PACAP KO	PACAP KO callus
WT				
WT callus				
PACAP KO				
PACAP KO callus				

Smad1 WB Day 7	WT	WT callus	PACAP KO	PACAP KO callus
WT				+
WT callus				+
PACAP KO				+
PACAP KO callus				

Smad1 WB Day 21	WT	WT callus	PACAP KO	PACAP KO callus
WT			+	+
WT callus			+	+
PACAP KO				
PACAP KO callus				
